# Supplementary material for: Computational models reveal how chloride dynamics determine the optimal distribution of inhibitory synapses to minimise dendritic excitability
Source: PLoS Comput Biol. 2022 Sep 23;18(9):e1010534. doi: 10.1371/journal.pcbi.1010534 (PMC9534446; doi:10.1371/journal.pcbi.1010534)
Supplement: Attachment — Submitted filename: Response to reviewers (Currin et al).docx [file pcbi.1010534.s001.docx]

Response to Reviewers

***Reviewer #1:*** *In this study, Currin et al., characterize how the location of GABAergic synapse on dendritic branches impact the ability of these synapses to regulate dendritic excitability quantified here by the metric termed “Inhibitory Level”. A major strength of this study is that it incorporates variable strengths of the GABAergic reversal potential and chloride dynamics which strongly impact GABAergic synapse. Overall, I am enthusiastic about this work, however, there are some concerns that should be addressed.*

We thank the reviewer for their enthusiasm for our work.

*Major Concerns*

*1. The schematic in Fig. 1 A is confusing and makes it hard to understand the setup of the simulated experiment. Because there are two electrodes drawn on the schematic, one darker than the other, and only one labeled with “excitatory input current” it appears that there is an excitatory input at 0.7X and a recording at 0.3X that is moving around to measure IL.*

Thank for this helpful suggestion, especially as this is the first figure and as such should be as intuitive as possible for the reader. On the reviewers suggestion we have now substantially altered the figure with the aim of improving understanding of our models.

*2. In the neuron schematic in Fig.1 A shows a somatic compartment connected to the left side of the dendrite however this is omitted from the schematics of the dendritic trees in the following figures. It is also unclear from the methods section if in your simulations the branch point (left end points) of your dendritic trees are capped or if they are connected to a somatic compartment or another dendritic compartment. The branch point should be connected to an additional compartment representing the soma or an additional dendritic compartment. Please clarify as these features will strongly impact model behavior such as the accumulation index and inhibitory level.*

We have followed the approach adopted by Gidon and Segev 2012 of having a small “parent” compartment for the branches to connect onto, which is much smaller (0.01 um in length). We have clarified this point in the methods section and have adjusted Fig1A to reflect this.

*3. In the introduction and discussion, the authors point out that dendrites possess active properties driven by NMDA, voltage-gated calcium, and voltage-gated sodium channels that add to the complexity of dendritic processing. This list should include voltage-gated potassium currents and hyperpolarization-activated cation currents. Inclusion of these additional currents is beyond the scope of the current study. However, it should be discussed how inclusion of these currents could impact the results of this study since the interaction between nonlinear conductances have been shown to have paradoxical effects on synaptic inputs (George, M. S., Nature neuroscience 2009).*

The reviewer makes an excellent point, we now include reference to voltage-gated potassium currents and hyperpolarization-activated cation currents in both the introduction and discussion. In addition, we have now added a new paragraph to the discussion where we discuss how the inclusion of non-linear dendritic conductances might interact with the results of this study. We also include reference to the George et al, Nature Neuroscience 2009 study.

*4. The finding that “multiple GABAergic synapses can cause greater inhibitory suppression at a shared branch junction than at any of the inhibitory synapses” is interesting and consistent with the effects demonstrated by Gidon Neuron 2012. Giddon et al. also showed that distal “off-path” rather than proximal “on-path” inhibition was more powerful dampening proximal excitable dendritic inputs and thus powerfully impacted the postsynaptic neurons output. However, in the current study, the inhibitory level on the proximal end of the dendrite (0.0 X) is the lowest when the inhibitory inputs are on the distal end of the dendrite (1.0 X), Fig.4B. These differences should be discussed.*

This is a nuanced point. Note that in Gidon and Segev 2012, where in Figure 1 and 2 they demonstrate that off-path inhibition can be more effective than on-path inhibition they use ‘Distance from “hotspot”’ as the x-axis. This is adjusting the inhibitory synapse location (i) while keeping the excitatory input stationary at a single hotspot location. This is not the same as our Fig. 4B where we have different inhibitory synapse locations (i) and for each of these inhibitory synapse locations we then test all possible excitatory input locations (d) (Note that elsewhere (Fig6) we adjust i, but the we also adjust d with i). Our top graph in Fig. 4B is analogous to Gidon and Segev 2012 Supplementary Figure S2B. You will note the similarity of these graphs, which are essentially the same despite being from 4 versus 6 branched structures respectively.

If one wanted to observe off-path versus on-path difference using our Fig. 4B (or Gidon and Segev 2012 S2B) a vertical slice should be chosen (e.g. a “hotspot” at 0.5 X) and then the lines at this slice should be traced back to see which Inhibitory synapse location (i) each line corresponds to. One would observe that indeed the lines from Inhibitory synapses that are a similar distance away from the “hotspot” but are off-path are higher (larger IL) than those that are from on-path inhibitory synapse locations. Essentially, our results are consistent with Gidon and Segev 2012, ie we don’t believe there are differences. We don't feel like we have anything to add to Gidon and Segev’s result of on-path vs off-path and so don’t discuss this, or focus on it in the paper.

*5. It should be discussed how these results may apply to glycinergic inhibition which should be less sensitive to Cl- fluctuations since these synapses do not flux HCO3 and therefore are more hyperpolarized. Should the distribution of glycinergic synapses be distributed differently than GABAergic synapses to optimize the dendritic inhibitory level?*

We have now added to the introduction to state that our results also apply to glycinergic inhibition as glycine receptors (GlyRs) are also primarily permeable to Cl-. However, we are confused by the reviewer’s suggestion that glycine receptors do not flux HCO_3_^-^. As far as we are aware this is definitely not the case. The ionic filters for GABA_A_Rs and GlyRs are very similar with very similar permeability values for HCO_3_^-^. This has been shown by the two systematic comparisons of HCO_3_^-^ permeability between the two receptor types that we are aware of:

1) Fatima-Shad, K., & Barry, P. H. (1993). Anion permeation in GABA- and glycine-gated channels of mammalian cultured hippocampal neurons. *Proceedings of the Royal Society of London. Series B: Biological Sciences*, *253*(1336), 69–75. https://doi.org/10.1098/rspb.1993.0083
2) Bormann, J., Hamill, O. P., & Sakmann, B. (1987). Mechanism of anion permeation through channels gated by glycine and gamma-aminobutyric acid in mouse cultured spinal neurones. *The Journal of Physiology*, *385*, 243–286.

*6. Why was the GABAergic reversal potential not calculated with the Goldman-Hodgkin-Katz voltage equation which is standard for calculating reversal potentials that are permeable to multiple ions?*

Thank you for raising this concern which was also shared by the 2^nd^ reviewer. This is a very important issue, which we had in fact thought deeply about and now realise that we should have clarified the necessity of our approach more clearly in the methods. Both reviewers are quite correct that the Goldman-Hodgkin-Katz (GHK) voltage equation for E_GABAA_ is certainly a more accurate method for calculating E_GABAA_ given any particular intracellular and extracellular concentrations of Cl^-^ and HCO_3_^-^ (see Figure 1A below). However, we would like to emphasize that we used the typical equivalent circuit, charge sum approach to modelling the dynamics of the membrane potential in dendritic compartments using the well-established NEURON simulation environment (Hines & Carnevale, 1997).  Here, in order to determine the change in voltage in each compartment at each time point, the membrane and axial currents are summed according to Kirchoff’s Current law where:

$$C_{m}\frac{dV_{m}}{dt}=-g_{pas}\left( V_{m}\left( t \right)-V_{rest} \right)-I_{syn}\left( t \right)+I_{axial}\left( t \right)+ I_{ext}\left( t \right)$$

(1)

In equivalent circuit models, synaptic currents are calculated using the following modified form of Ohm’s law:

$$I_{syn}=g_{syn}\underset{driving force}{\underbrace{\left( V_{m}-E_{syn} \right)}}$$

(2)

In our work, of central importance was calculating I_GABA_ for use in Equation 1, but also critically, the chloride current I_Cl_ needed to be calculated so that intracellular Cl^-^ concentrations in each compartment, and then E_GABA_, could be updated accordingly (ie we modelled dynamic Cl^-^). As GABA_A_Rs are permeable to both Cl^-^ and HCO_3_^-^, I_GABA_ is combination of both I_Cl_ and I_HCO3_. One option (which we believe the reviewers are suggesting) is that we use the following formula to calculate I_GABAA_:

$$I_{GABA}=g_{GABA}\underset{driving force}{\underbrace{\left( V_{m}-E_{GABA} \right)}}$$

(3)

And use the GHK voltage equation (below) to calculate E_GABA_

$$E_{GABA}=\frac{R T}{F}\log\left( \frac{\frac{4}{5}\left[ Cl^{-} \right]_{i}+\frac{1}{5}\left[ HCO_{3}^{-} \right]_{i}}{\frac{4}{5}\left[ Cl^{-} \right]_{o}+\frac{1}{5}\left[ HCO_{3}^{-} \right]_{o}} \right)$$

(4)

Then presumably I_Cl_ would be calculated as 4/5*I_GABA_? This approach would be erroneous, as although the relative conductances of GABA_A_Rs to Cl^-^ and HCO_3_^-^ are fixed (4:1), the currents for Cl^-^ and HCO_3_^-^ are not a fixed proportion and will change depending on their respective reversal potentials.

Consider the case when Vm ≈ E_GABA_ (say GABA_A_Rs have been very strongly activated shifting the V_m_ close to E_GABAA_), then according to the above calculation I_GABAA_ ≈ 0 and hence I_Cl_ would also be 0 and despite further GABA_A_R activation, Cl^-^ loading would not occur and E_GABAA_ would not continue to shift positively (see Figure 1B below). This is not what has been consistently observed experimentally (Kaila et al., 1997; K. Staley et al., 1995; K. J. Staley & Proctor, 1999). For example intense GABA_A_R activation alone causes biphasic GABA potentials, where by the initial response starts as hyperpolarising but then shifts to depolarising. This is a well characterised phenomenon, please see the following for a review we have written on this topic (Raimondo et al., 2012). Therefore to faithfully capture ionic plasticity at GABA_A_R synapses (ie Cl^-^ dynamics) we calculated I_Cl_ and I_HCO3_ separately given their independent and differentially dynamic reversal potentials but we fixed their relative conductances at 4:1. We therefore used the following formulation.

$$I_{GABA}=I_{Cl^{-}}+I_{HCO_{3}^{-}}$$

and

$$I_{Cl^{-}}=\frac{4}{5} g_{GABA}\left( V_{m}-E_{Cl^{-}} \right)$$

$$I_{HCO_{3}^{-}}=\frac{1}{5} g_{GABA_{A}}\left( V_{m}-E_{HCO_{3}^{-}} \right)$$

(5)

It is important to note that in the figures in our manuscript E_GABAA_ is plotted purely as a readout (it is not used to calculate the I_GABAA_ or I_Cl_ as described above). Therefore we believe it is most representative to calculate E_GABAA_ for the purposes of our figures using:

$$E_{GABA}=\frac{4}{5}E_{Cl^{-}}+\frac{1}{5}E_{HCO_{3}^{-}}$$

(6)

This better represents the dynamics of the constituent currents that NEURON calculates. We hope this reasoning makes sense. We have now added to the methods section where we justify this approach.

**Figure 1: The GABA_A_R reversal potential (EGABA) and approaches for modelling GABA_A_R currents**

$$E_{GABA}= \frac{4}{5} E_{Cl^{-}} + \frac{1}{5} E_{HCO_{3}^{-}}$$

$$E_{GABA}=\frac{R T}{F}\log\left( \frac{\frac{4}{5}\left[ Cl^{-} \right]_{i}+\frac{1}{5}\left[ HCO_{3}^{-} \right]_{i}}{\frac{4}{5}\left[ Cl^{-} \right]_{o}+\frac{1}{5}\left[ HCO_{3}^{-} \right]_{o}} \right)$$

(**A**) E_GABA_ can be calculated by combining the separately calculated reversal potentials of Cl^-^ and HCO_3_^-^ (“Ohmic”) or the Goldman-Hodgkin-Katz (GHK) equation, according to the receptor’s permeabilities to Cl^-^ and HCO_3_^-^. (**B**) We simulated intense GABA_A_R activation with a 2 Hz activation of a GABA_A_Rs (g_GABA_ = 900 nS, τ_GABA_ = 8 ms) in a single compartment model. Note that when using the Ohmic equation (blue line), GABA_A_R potentials shifted from being hyperpolarsing to depolarsing, which was driven by Cl- loading. However, when the GHK voltage equation (grey line) approach was used GABA potentials remained hyperpolarising and Cl- loading was minimal. Inset left, the hyperpolarising postsynaptic response of GABA_A_R activation for both Ohmic and GHK equations. Right inset, using Ohmic approach yields a depolarising postsynaptic response after continued GABA_A_R activation, but the GHK equation approach is still hyperpolarising, which does not represent experimental data for sustained GABA_A_R activation (Staley et al., 1995).

We do acknowledge that an alternative approaches could be used. One could abandon the equivalent circuit framework (and not use NEURON) and instead use the Goldman-Hodgkin-Katz (GHK) flux density/current equation to calculate Cl^-^ and HCO_3_^-^ currents via GABA_A_Rs (Johnston & Wu, 1995). This would then be consistent with E_GABAA_ calculated using the GHK voltage equation. However, for consistency one would then need to use this equation for all the passive/ leak currents as well (not just currents via GABA_A_RS), which would mean separating them into their ionic constituents. One should also then use the Nernst Planck Equation to model electrodiffusion between compartments (and not Ohms law as is used by the NEURON environment) (Johnston & Wu, 1995). The charge difference approach could be used to calculate membrane voltage in each compartment (Fraser & Huang, 2004). However, each of these adaptions would be prohibitively complex and too computationally demanding to perform the simulations/ investigations we have performed here. We have now added a paragraph to the discussion where we discuss the potential limitations of our modelling approach and describe how with improved access to computational resources, enhancements to our models could be considered.

References for this response:

Fraser, J. A., & Huang, C. L.-H. (2004). A quantitative analysis of cell volume and resting potential determination and regulation in excitable cells. *The Journal of Physiology*, *559*(Pt 2), 459–478. https://doi.org/10.1113/jphysiol.2004.065706

Hines, M. L., & Carnevale, N. T. (1997). The NEURON simulation environment. *Neural Comp.*, *9*(6), 1179–1209. http://www.mitpressjournals.org/doi/pdf/10.1162/neco.1997.9.6.1179

Johnston, D., & Wu, S. M. (1995). *Foundations of cellular neurophysiology*. 676.

Kaila, K., Lamsa, K., Smirnov, S., Taira, T., & Voipio, J. (1997). Long-Lasting GABA-Mediated Depolarization Evoked by High-Frequency Stimulation in Pyramidal Neurons of Rat Hippocampal Slice Is Attributable to a Network-Driven, Bicarbonate-Dependent K+ Transient. *J. Neurosci.*, *17*(20), 7662–7672. http://www.jneurosci.org/content/17/20/7662.full

Raimondo, J. v., Markram, H., & Akerman, C. J. (2012). Short-term ionic plasticity at GABAergic synapses. *Front. Synaptic Neurosci.*, *4*(October), 1–9. https://doi.org/10.3389/fnsyn.2012.00005

Staley, K. J., & Proctor, W. R. (1999). Modulation of mammalian dendritic GABA(A) receptor function by the kinetics of Cl- and HCO3- transport. *The Journal of Physiology*, *519*(1999), 693–712. https://doi.org/PHY_9331 [pii]

Staley, K., Soldo, B., & Proctor, W. (1995). Ionic mechanisms of neuronal excitation by inhibitory GABAA receptors. *Science*, *269*(5226), 977–981. https://doi.org/10.1126/science.7638623

*Minor Concerns*

*1. Fig. 1C. It is difficult to tell that there are three overlapping curves in C and the light gray dashed lines that show IL every 5ms are very hard to see.*

We have now substantially revised Fig 1 and have taken this into consideration.

*2. In Fig.3 are the curves in panel E supposed to be color coded with panels B-D?*

Indeed they are, we have now added a legend to panel E for clarity.

*3. In Fig.3 the term “effective number of synapses” in the caption but “effective number of branches” in the figure labels.*

Thank you for alerting us to this, for consistency we now refer exclusively to “effective number of synapses”.

*4. Figure 4. Is the label “Inhibitory level” that is between the color bar for panel A and the x axis in panel B a shared label?*

It was intended to be a shared label, yes. We have now used individual labels for clarity.

*5. Fig.4 F. The colors are a little hard to distinguish between the curves for the tree, branch and focal distributions.*

We now use a new colour scheme which we hope makes distinguishing the various curves easier.

*6. Doyon et al., Frontiers in Cellular Neuroscience 2016, and Phillips et al., eLife 2020 also looked at how Cl- dynamics impact GABAergic inhibition and should be cited.*

We agree with the reviewer and now cite these two relevant papers.

***Reviewer #2:*** *In their manuscript entitled “Computational models reveal how chloride dynamics determine the optimal distribution of inhibitory synapses to minimise dendritic excitability” Christopher Currin and Joseph Raimondo presented a study which ought to identify the optimal distribution of inhibitory synapses within dendritic trees. The authors use a purely computational approach with a simple geometric topology and passive membrane properties. Their main results are that 1.) for 4 and more dendrites with equidistant synapses the local inhibition maximizes at the junction of the dendrites if more that 50% of the dendrites have active inhibitory synapses, that 2.) the level of inhibition superimposes at these junctions with a higher effectivity for hyperpolarizing inhibitory synapses, that 3.) for hyperpolarizing synapses a lower occupancy with active synapses is required for a junctional superimposed inhibition, and 4.) that under consideration of dynamic EGABA the optimal position of inhibitory synapses is about 6-9% of the dendritic length distally to the junction (depending on the number of dendrites).*

*The question how GABAergic synaptic inputs interacts within a dendritic tree is an important question. The approach to address this question by using a passive dendrite to isolate the general principles of dendritic integration within a dendrite is a reasonable methodology. This study is a significant expansion of basic principles published by Gidon & Segev (2012) and as such can provide new important information about the basic principles underlying the interaction between structure and function of neurons. The implementation of static and dynamic EGABA to identify the optimal localization of inhibitory synapses provides important additional information to comprehend the strategic localization of synapses for efficient inhibitory control. In this respect the use of the simplified models is required to understand the basic principles underlying the principles of interaction between inhibitory synapses. The models and each experimental step are clearly described and nicely illustrated. In particular, I appreciated how the authors linked in the discussion their theoretical assumptions with recent data on the microanatomical distribution of GABAergic inputs.*

*However, I my opinion in particular the simple geometric structure of the used dendrite model critically limits the transfer of the main finding to a more realistic situation. The limitations of the simplified models, which as mentioned is an important aspect of this study, may be addressed by some exemplary simulations and must be discussed in detail. And finally, there are some small inaccuracies that should be corrected. A consideration of these point may enhance the applicability of this excellent manuscript.*

*Major:
1.) Topology of the dendritic aggregates:*

*I have severe concerns that the construction/implementation of your dendritic aggregates may underlie to some extend your main finding that “… the optimal distribution of available GABAergic synapses to maximise local inhibition in a branched dendritic tree is to place synapses surrounding, but not at, a branch junction (Line 514-516). In line 421 you state that “.. each inhibitory synapse [contribute] to a highly pooled Cl- load” thereby enhancing the positive shift of EGABA. However, this observation was most probably caused by the rather artificial topology of your dendritic aggregate, with n dendrites of equal function. Only in this case four GABA synapses can co-localize incrementally close to a common junction. Figure 4E of the Gidon & Segev (2012) depicts the geometric organization of your dendrite arrangement probably more honestly. With this picture in mind, in particularly the fast breakdown of EGABA for synapses close to the junction became more obvious. Regarding the normal dendritic topology, this is, however, a rather artificial situation, as in your case 4 equal dendrites merged without an additional "main dendritic branch" connecting them to the soma (and allowing electrotonic spread and, more important here, diffusion of Cl- ions). Therefore, I would strongly suggest to investigate whether the “optimal inhibitory synapse position” was still observed at the branch point if a diffusional Cl- sink exists in one of the dendrites (which has no synapse).*

We thank the reviewer for raising these valid concerns. Regarding the topologies, we have now added a new panel Figure 2Ai, which we hope provides conceptual clarity as to the dendritic arrangements / geometric organizations our topologies represent (including that shown in Figure 4E of Gidon & Segev, 2012 . That is, all the geometric organizations we show in Figure 2Ai are considered equivalent in our modelling framework. We then use one of these geometric organizations to represent a branched dendritic structure for the remainder of the paper.

We also agree with the reviewer that a Cl^-^ sink (for example provided by an additional “parent” dendritic branch) would strongly affect our results. Therefore we now provide new data from new numerical simulations where we use topologies where we have added a Cl^-^ sink in the form of an additional parent dendritic branch devoid of inhibitory synapses. This is presented as a new figure (Figure 8). We investigated the optimal location for the inhibitory synapses on the “child” branches (that is, where IL is greatest) with a parent branch of different diameters (ie different sized Cl^-^ sinks) and in cases where there was a proportional length change or not (to keep the length constant the same or not). We see a diameter-dependent change in optimal inhibitory synapse placement, ie the bigger the Cl^-^ sink, the closer inhibitory synapses may be placed to the shared branch junction. We have also added a sentence to the discussion where we state how the presence of Cl^-^ sinks can ameliorate the effects of Cl^-^ loading.

*2.) Quantification of the inhibition level:*

*At the first sight it was not clear to me, how you can get an IL of 7.5 in Fig. 2Bv. An IL of 1 meant (according to your definition), that the inhibitory synapse eliminated all "excitatory" membrane responses. As soon as you shift to a sufficient hyperpolarization the voltage integral of the excitation+inhibition become smaller (i.e. more negative) and the control integral and thus the numerator in formula #1 becomes larger than the denominator. However, for a IL level of 7.5 the absolute voltage integral with inhibition must be 8.5 times larger than for the control interval (with only excitation). But from Fig. 1B I assume that you depolarized potential is in the range of ca 1 mV. However, if the inhibition abolishes all depolarizing EPSPs and clamps Em to ECl, this means that Em can be maximal 1 mV hyperpolarized to the RMP (resulting in an IL of 2). The most probable explanation I could find is that the total Ri of the dendrite aggregate decreases which each added dendrite, leading to diminished Vd amplitudes under control conditions (which will make the ratio between the GABAergic hyperpolarization and the control depolarization larger). I would suggest to illustrate this interpretation (if it is true) with the voltage responses obtained in multi dendrite aggregates. I wonder whether this diminished voltage response can/must be compensated by increased injection current (e.g. to make the “accumulation index” comparable between conditions with different numbers of dendrites). At least you should discuss how to disentangle the “passive inhibitory effect” from the more elaborated dendritic tree, from the amount of synaptic inhibitory efficacy.*

The reviewer’s interpretation is indeed correct in that the initial depolarisation during “control” Vd is lower with more branches. This is the identical approach used by Gidon and Segev 2012. To make this approach more explicit. We have now amended Figure 2A,B, with new inserts showing the voltage traces for the different number of branches and both ∇EGABA=0 and ∇EGABA=-1.
The idea of an increased compensatory excitatory current injection depending on the different number of branches is an interesting, but different question, and would also be not trivial to implement. (e.g. whilst “total” Ri is indeed lower for aggregates with more branches, Ri is also different at each point of the dendrites, how would one choose or scale the excitatory current injection appropriately?) In this case, the compensatory excitatory input current would not be identical and so comparisons within and between branch aggregates would be difficult to interpret.

*3.) Calculation of EGABA:*

*The formula provided in line 145 is wrong and cannot be used to calculate EGABA. You have to use the GHK. You can easily test this, eg. with an [HCO3-]i of 12.165481 mM (providing an EHCO3- of -18 mV at 310.15 K and a [HCO3-]e of 24 mM) there is a clear deviation between the GHK-Potential and your simplified formula (e.g. -72.9 vs -71.0 mV at 5 mM Cl; -60.1 vs -56.2 mV at 10 mM Cl-; -51.5 vs -47.7 mV at 15 mM Cl-). Note that the GHK can be interpreted as the "contribution" of the HCO3- permeability depending on the Cl- concentration. Please see Farant & Kaila (2007) for a detailed description of this relation.*

Please see our detailed response to this point in our response to Reviewer 1, who had the same concern.

*4.) Restriction to stationary current:*

*One conceptual problem I have with your study is the IL was determined by stationary currents and thus the temporal aspects, in particular the effects of gGABA on the membrane time constant, are neglected. Can you at least speculate how this parameter will affect the optimal localization of inhibitory synapses?*

We agree that the temporal aspects of input are an important consideration which we did not explore within our framework. Gidon and Segev 2012, extended their analysis to explore the effects of transient GABAergic inputs, see their supplementary material (Figure S8 and S9). We do not believe that it was within the scope of our manuscript to try and extend their already excellent analysis in this regard. Therefore, we have added a sentence to the end of the first results section that points the reader to the time-varying input work of Gidon and Segev. The effects of Cl^-^ dynamics and Cl^-^ loading become more apparent with longer periods of inhibitory synaptic drive, and conversely are less important for very short periods of inhibitory input. We now explicitly state this in the discussion. That is, for very brief duration inhibitory synaptic input, the optimal location of inhibitory synapses to maximise IL would be closer to the junction as there would not be time for Cl^-^ loading to occur.

*5.) Relevance of the first results subchapter:*

*To be strict, I do not really see how several details of this subchapter (Line 169-200) contributes to the overall results of the manuscript. You may consider to eliminate most algebraic terms from here and just define the IL metrics (in accordance to formula #1). In line 198 you state that “Eg. 1 […] captures both conductance and IPSP effects of inhibition”, however, this formula in the present form just includes voltage differences. In addition, I have a problem with the statement “Eq. 3 can be calculated analytically” (line 195). If I understand all parameters correctly, you need the experimentally determined voltage attenuation Ai,d (and Ad,i) for this, which is actually given by [(Vd-Vd,i)/Vd]. In case you decide to keep this paragraph, I would suggest to slightly change the indices in Eq.3. The use of gi and Ri is a little misleading as gi implies this is the membrane conductance at the position i. It would be clearer if you indicate already in the formula that gi is referring to the GABAeric conductance. And finally, the red stricken variables in the derivation of Eq. 3 look weird, like forgotten deleted items in the track-change mode of word. I wonder whether it is necessary to provide algebraic operations in this way in a manuscript.*

We thank the reviewer for their detailed and helpful suggestions which we have now implemented. We have now removed some of the algebraic terms and the derivation for Eq. 3. We have changed gi to gGABAi as suggested (also in Figure 1). We have also removed the red stricken variables as requested. Regarding capturing conductance and IPSP effects, we now add a sentence stating that the effects can be disentangled using Eq 2. For determining voltage attenuation, we have added a statement that this can be calculated using Rall’s cable equations (Rall, 1959) [Gidon and Segev also have more detail on this in their main paper and extended this to multiple dendrites in their supplementary material].

*6.) Impact of the results on active dendrites:*

*While I highly appreciate you approach to investigate the interaction between inhibitory synapses in a strictly dendro-centric approach, at the end I’m wondering how your findings will be affected by active properties of the dendrites. I would appreciate if you can include some exemplary experiments in the manuscript which demonstrate whether the inclusion of several active dendritic features will augment or ameliorate your findings. At least, you should discuss the impact of your findings for active dendrites.*

We agree with the reviewer that it would be useful to consider how our finding would be affected by active properties of dendrites. Although we believe that further simulations involving active dendrites would be beyond the scope of this manuscript, we now include a new paragraph in the discussion where we discuss the impact of our findings for active dendrites.

Minor:

*Line 19: While it became clear to me, what “accumulation […] of inhibitory effectiveness” means when reading the full text, this statement was really puzzling to me when first reading the abstract. Maybe it is possible to find a description for this observation that it easier to understand in the abstract.*

We have used the sentence,

We found that the inhibitory effectiveness of dendritic GABAergic synapses **combines** at an encircled branch junction, “inhibitory accumulation”.

in the abstract to hopefully get the point across a bit more clearly.

*Line 49: I would describe a dendrite, which is a physical entity of the neuron, more as a "neuronal part", "neuronal compartment" or "neuronal domain" rather than a "feature" of a neuron.*

We agree and have chosen the better-suited and well-suggested “neuronal domain”.

*Line 75: In my humble opinion there are better references for the dominance of Cl- permeability in GABA(A) receptors available than these experiments in the crayfish muscle.*

While we acknowledge the reviewers point, we fear that the discoverer of bicarbonate permeability of GABAARs – using crayfish muscle - Kai Kaila, might disagree. In a similar vein one could equally argue that there are better references for describing the mechanisms underlying action potential generation than Hodgkin and Huxley’s seminal work because these were performed using the squid giant axon? Nonetheless, along the reviewer’s suggestion we have now added additional references where experiments were performed in mouse neurons.

*Line 85-102: Within this paragraph you used “synapses” and “inhibitory synapses” intermingled and synonymously. However, as in many studies inhibition was quantified by the influence of GABA on excitatory synapses, it was at first reading in some sentences ambiguous to me whether “synapse” may also refer to excitatory “test” synapses here. Therefore, I would suggest to use the phrase “inhibitory synapses” throughout this para.*

Thank you for this suggestion, we have now made sure to use “inhibitory synapses” throughout this paragraph.

*Line 134 “50 ms otherwise”: In line 180 you mention that you determine IL for an integral from 0-150ms. Please specify*.

We have now clarified that we used a 5 ms time integration window from 145 ms until 150 ms.

*Line 179 “Because the neuronal membrane is a capacitor and IL is calculated from Vm, the IL was taken at 150 ms unless otherwise stated”. To my understanding, this is not a valid explanation to choose exactly 150 ms as upper boarder for IL determination. Is there anything special with this time point regarding e.g. the time constant of the dendritic membrane or the stability of deltaEm or IL*?

We did not perform extensive steady-state analysis but observed that by 100 ms the IL had *largely* reached steady-state and by 200 ms was definitively at steady-state (see insets of Figure 1 and Figure 2). Choosing 150 ms was a good compromise between minimising computational load and maintaining accuracy.

*Line 218 and Fig. 2B, “NablaEGABA = -1 mV”: According to the definition in Formula #1 (line 122: nablaEGABA = VRest - EGABA) an nablaEGABA of -1mV means that EGABA was 1 mV larger (it is shifted more in the positive/depolarizing direction) than VRest.*

Thank you for noticing this, we have now updated the equation to

∇EGABA = EGABA – VRest

So -1 = -66 - - 65

*Fig. 2D: In my pdf the colors of the 8 and 16 branch data look identical. Consider to use different colors, in this panel displaying only the shading information would also be sufficient.*

We have now adjusted the colour of the 16 branched dendrite throughout the manuscript.

*Line 232 (Fig. 2D) “…AccIdx increased linearly…”: Are these indeed linear functions with different slopes for NablaEGABA of (-)1mV and (-)2 mV? Or are these slight deviations from a linear dependency that became apparent only for larger dendrite numbers?*

The reviewer is quite correct. We have now qualified the sentence to read “approximately linearly”.

*Line 261-263, Fig. 3B: Here I like to know whether this interesting result is just caused by the mere amount of gGABA in adjacent position. In my opinion a more elusive approach would be to enhance the number of synapses in the “non-analyzed” branches and keep only 1 synapse in the analyzed branch. Does the results differ between one n x gGABA synapse in one adjacent dendrite and n gGABA synapses in n adjacent dendrites.*

The IL in branches that have no inhibitory synapses (or fewer synapses) are indeed due to the gGABA of the synapses on the other branches in the dendrite. Perhaps the insets in Figure 3 C and D as well as E and F partly speak to the “elusive approach” envisioned by the reviewer? In E and F, all the branches are analyzed (ie the IL values as a function of distance from the junction are displayed for all branches) and the effective number of synapses of 175% means that for the 4 branched dendrite, one branch has only 1 synapse and the others have 2.

*Line 265 “In addition, when some branches had more synapses than others (e.g. 150% effective number of synapses), the IL0 was naturally shared between them, but the ILd=i differed.”: In my opinion this sentence sounds strange, at it is obvious that ILd=i must differ (because gGABA differs between both sites). Consider rephrasing.*

We have now rephrased this sentence and hope that it sounds less strange.

*Line 270: Theoretically there should be no “saturation”, but an approximation towards hardly detectable voltage deflections if gGABA >> gInput. Consider rephrasing.*

We have now added a clarifying sentence indicating such an approximation

*Line 283 “The branches without synapses ..”: Is this really "without synapses" or "without extra synapses"? From Fig. 3F-inset I assume that this should be “extra synapses”.*

We have now corrected this.

*Line 314: Here you mention that more than 4 dendrites are required for an accumulation of IL at the junction, which is in contrast to Gidon & Segev (2012), who found that at least 3 inhibitory synapses are required for such an elevated inhibition. As long as you didn't test 3 branches, you should state here more carfully "but not in 2 branches".*

We did not test 3 branches so in line with the reviewer’s suggestion we have now worded this sentence (and a few others) more carefully.

*Line 339, Fig. 4E: Here the color code suggested that the IL profile in the branches without synapses appeared to be identical in the "focal" and the "branch" situation. As this observation might have some functional impact, I wonder whether you already quantified whether such identical IL profiles indeed can be observed.*

We have not explicitly looked at the “silent branches” but there does appear to be a difference in silent branches between the “focal” and “branch” distributions that is proportionally the same (i.e. absolute differences scale) with ∇EGABA and more noticeable differences with fewer number of branches. We have included an image here for the reviewer’s interest (left is 8 branches and right is 4 branches, rows are -5, -2, and 0 ∇EGABA).

*Line 356 “up until now”: Consider to be more specific here. E.g. "in Figs. 1-4" ?*

Thank you for this suggestion, which we have implemented.

*Line 368: In you experiments it appears that EGABA approaches local values of -65 mV (Fig. 5D), suggesting that the transmembrane Cl- gradient breaks down. Does this happen under physiological conditions or is this just caused by the artificially long and strong GABAergic activity in your experimental paradigm. Please comment/discuss*.
This has been observed experimentally (Kaila et al., 1997; K. Staley et al., 1995; K. J. Staley & Proctor, 1999) including with high-frequency input such as in seizures (@Joe, which refs you think most relevant? Richard’s Brain paper? A paper from Andy’s group like Hannah’s?)

*Line 401: Please consider rephrasing to emphasize that the maximum IL was obtained at X=0.5.*

We have now this accordingly.

*Line 446 “the greatest site of IL was…”. Consider rephrasing to "the site of greatest IL effect was …"*

We have rephrased to “the site of greatest inhibitory effect”.

*Line 450: Here you state that the “maximum level of IL” was reached at ca. 0.08X, whereas in line 415 an optimal IL distances of 0.07x was given (both for 4 dendrites and a EMFGABA of -5 mV). Please check and elaborate the description if there is an experimental difference between both simulations*.

The difference is that in Figure 6, IL was recorded at the site of the inhibitory synapse (IL_d=i_), so the greatest was 0.07 X. In comparison, Figure 7 also shows the IL at the junction (IL_0_), which in this case is greater than at the synapse (IL_d=i_) and the highest at 0.08 X. We have now added a sentence to this paragraph with the aim of clarifying this.

*Line 480 “We find that GABAergic synapses with more negative EGABA are considerably better at suppressing local dendritic excitation (increased IL).” In my opinion this observation is probably not the main fining of your study (and also not a new and unexpected one). Therefore, this finding is probably not the best result to emphasize in the first sentences of the discussion. Consider to rephrase this section*.

Thank you for this suggestion. We agree with the reviewer in saying that more negative EGABA is predicted to be better (not novel), but the extent to which it is better (i.e. multiplicatively) and that it is impactful everywhere on the dendrite we believe is worth emphasizing. We have now amended the sentence to read (bold for important bits we have changed). “We find that GABAergic synapses with more negative EGABA are **multiplicatively** better at suppressing local dendritic excitation **throughout the branches** (increased IL)”

*Line 507 “strongly affect IL and should be considered under a different framework that includes the time component of [Cl-]i loading (Eq. 1).” Please note that Eg. 1 did not explicitly include Cl- or IPSP*.

For simplicity we have now removed this sentence.

*Line 512: “…as the precise morphological structure (volume, surface area, compartmental ion differences, etc.)”. “Compartmental ion differences” are not really a morphological structure, but you may add other morphological features likes spines or tortuosity here.*

We have added your suggestions and separated compartmental ion differences to not be associated with the morphological structure per se.

*Line 578 “… is no “V” marker….”: As you used the Nabla for identifying the EMF(GABA) I would suggest to use here the term "arrowhead" to avoid confusion.*

We have used the reviewer’s suggestion.

*Line 662: Please correct typo “placement”.*

This has been corrected.

*Fig. 7A, right panel. Here it is not clear to me how to interpret this graph. In the "branch" situation all synapses should distribute evenly along the dendrite, so which parameter is plotted here on the x axis and how can the complicated shape of the location dependency be explained?*

We have now added text in the relevant results section to try explain the location dependency of the “branch” distribution.

*Reference 31: Please correct “excitation in CA1 pyramidal neurons”.*

We have corrected this mistake.
