# Supplementary material for: Computational models reveal how chloride dynamics determine the optimal distribution of inhibitory synapses to minimise dendritic excitability
Source: PLoS Comput Biol. 2022 Sep 23;18(9):e1010534. doi: 10.1371/journal.pcbi.1010534 (PMC9534446; doi:10.1371/journal.pcbi.1010534)
Supplement: Attachment — Submitted filename: Currin etal review_2.docx [file pcbi.1010534.s002.docx]

The authors have addressed most of the reviewer comments in the revised manuscript. However, a couple of issues remain.

**Major Comment**

**1) EGABA calculation.** Although hard to see on the scale given in Figure 1A of the response document, the difference between GHK and Ohmic calculation of the GABAergic is quite large. About 5mV when [Cl-]_i=5.0mM judging from panel B at t=0s. Additionally, the response/analysis provided regarding the calculating of EGABA and Cl- dynamics with the GHK formulation is not correct because the portion of the GABAergic current carried by Cl- ions is not simply 4/5*I_GABA_. Instead, I believe it should be:

$$I_{Cl}=\chi\cdot g_{GABA}\cdot\left( V_{m}-E_{Cl} \right)$$

Where $\chi$ represents the fraction of the total GABAergic current carried by Cl- ions and is given by:

$$\chi=\frac{\left( E_{HCO3}-E_{GABA} \right)}{\left( E_{HCO3}-E_{Cl} \right)}.$$

This has been used in previous modeling papers that simulate Cl- dynamics and $\chi$ can be calculated by letting the total GABAergic current equal some ratio of a Cl and HCO3 current.

$$I_{GABA}={\chi\cdot I}_{Cl}+\left( 1-\chi\right)\cdot I_{HCO3}$$

$$\downarrow$$

$$g_{GABA}\cdot\left( V_{m}-E_{GABA} \right)=\chi\cdot g_{GABA}\cdot\left( V_{m}-E_{Cl} \right)+\left( 1-\chi\right){\cdot g}_{GABA}\cdot\left( V_{m}-E_{HCO3} \right)$$

$$\downarrow$$

$$-E_{GABA}=-\chi\cdot E_{Cl}+-E_{HCO3}+{\chi\cdot E}_{HCO3}$$

$$\downarrow$$

$$\chi=\frac{\left( E_{HCO3}-E_{GABA} \right)}{\left( E_{HCO3}-E_{Cl} \right)}$$

It’s my view that the simulations with chloride dynamics should be rerun using the GHK equation to calculate the GABAergic reversal potential and the scaling factor $\chi$ rather than 4/5.

**Minor comment**

1) Fig 1
   a. The figure legend for panel B says that the IL traces are red on the left and orange on the right but in my version of the pdf these look like they are only slightly different in color.
   b. Legend text for panel C needs to be updated as it references a green dotted, dashed brown and purple lines that are no longer included in this version of the figure.
