# Supplementary material for: Computational models reveal how chloride dynamics determine the optimal distribution of inhibitory synapses to minimise dendritic excitability
Source: PLoS Comput Biol. 2022 Sep 23;18(9):e1010534. doi: 10.1371/journal.pcbi.1010534 (PMC9534446; doi:10.1371/journal.pcbi.1010534)
Supplement: Attachment — Submitted filename: Response to reviewers round 2.docx [file pcbi.1010534.s003.docx]

We thank the reviewers for carefully considering how to model chloride currents as accurately as possible. We previously were not aware of models that used this formulation. In truth, we struggled to find papers that modelled chloride in this manner. Regardless, we thought this was an elegant solution and have re-run all simulations with chloride dynamics. Although specific values have changed slightly, the conclusions remain the same.

As a minor note, the Branch panel in figure 7 has been updated. Although we ran simulations with different spacing, the graphs were hard to interpret and introduced the new concept of spacing. We believe the current version is more interpretable and in-line with a previous figure (Fig. 4).

*Reviewer #1: The authors have addressed most of the reviewer comments in the revised manuscript. However, a couple of issues remain.*

***Major Comment***

**1*) EGABA calculation.*** *Although hard to see on the scale given in Figure 1A of the response document, the difference between GHK and Ohmic calculation of the GABAergic is quite large. About 5mV when [Cl-]_i=5.0mM judging from panel B at t=0s. Additionally, the response/analysis provided regarding the calculating of EGABA and Cl- dynamics with the GHK formulation is not correct because the portion of the GABAergic current carried by Cl- ions is not simply 4/5*I_GABA_. Instead, I believe it should be:*

$$I_{Cl}=\chi\cdot g_{GABA}\cdot\left( V_{m}-E_{Cl} \right)$$

*Where* $\chi$ *represents the fraction of the total GABAergic current carried by Cl- ions and is given by:*

$$\chi=\frac{\left( E_{HCO3}-E_{GABA} \right)}{\left( E_{HCO3}-E_{Cl} \right)}.$$

*This has been used in previous modeling papers that simulate Cl- dynamics and* $\chi$ *can be calculated by letting the total GABAergic current equal some ratio of a Cl and HCO3 current.*

$$I_{GABA}={\chi\cdot I}_{Cl}+\left( 1-\chi\right)\cdot I_{HCO3}$$

$$\downarrow$$

$$g_{GABA}\cdot\left( V_{m}-E_{GABA} \right)=\chi\cdot g_{GABA}\cdot\left( V_{m}-E_{Cl} \right)+\left( 1-\chi\right){\cdot g}_{GABA}\cdot\left( V_{m}-E_{HCO3} \right)$$

$$\downarrow$$

$$-E_{GABA}=-\chi\cdot E_{Cl}+-E_{HCO3}+{\chi\cdot E}_{HCO3}$$

$$\downarrow$$

$$\chi=\frac{\left( E_{HCO3}-E_{GABA} \right)}{\left( E_{HCO3}-E_{Cl} \right)}$$

*It’s my view that the simulations with chloride dynamics should be rerun using the GHK equation to calculate the GABAergic reversal potential and the scaling factor* $\chi$ *rather than 4/5.*

We have re-run all simulations with chloride dynamics and updated the text accordingly.

***Minor comment***

1) *Fig 1*
   a. *The figure legend for panel B says that the IL traces are red on the left and orange on the right but in my version of the pdf these look like they are only slightly different in color.*
   b. *Legend text for panel C needs to be updated as it references a green dotted, dashed brown and purple lines that are no longer included in this version of the figure.*

These have been corrected, thanks for pointing them out!

*Reviewer #2: I appreciate that the authors considered all of my comments and provided more than sufficient responses.

Regarding the computation of Cl- and HCO3- dependency of reversal potentials and ion fluxes via GABA receptors, there are solutions and models in the NEURON environment available. However, I agree with the authors that their simplified solution is perfectly suited for their questions.*

In line with the suggestions of reviewer 1, we have used their suggested dynamics.

*Line 20. Here the term "inhibitory accumulations" in citation marks reads strange. In my humble opinion it would be sufficient to state in the next sentence (line 21) "**The extend of this inhibitory accumulation ...".*

We have made this suggested change.
